# Cholera Outbreak Linked with Lack of Safe Water Supply Following a Tropical Cyclone in Pondicherry, India, 2012

**Published:** 2015-03

**Authors:** Tony Fredrick, Manickam Ponnaiah, Manoj V. Murhekar, Yuvaraj Jayaraman, Joseph K. David, Selvaraj Vadivoo, Vasna Joshua

**Affiliations:** ICMR School of Public Health, National Institute of Epidemiology, Indian Council of Medical Research, TNHB, Ayapakkam, Chennai 600 077, India

**Keywords:** Cholera, Outbreak, Post-cyclone, India

## Abstract

In the aftermath of a severe cyclonic storm on 7 January 2012, a cluster of acute diarrhoea cases was reported from two localities in Pondicherry, Southern India. We investigated the outbreak to identify causes and recommend control measures. We defined a case as occurrence of diarrhoea of more than three loose stools per day with or without vomiting in a resident of affected areas during 6-18 January 2012. We used active (door-to-door survey) and stimulated passive (healthy facility-based) surveillance to identify cases. We described the outbreak by time, place, and person. We compared the case-patients with up to three controls without any apparent signs and symptoms of diarrhoea and matched for age, gender, and neighbourhood. We calculated matched odds ratio (MOR), 95% confidence intervals (CI), and population attributable fractions (PAF). We collected rectal swabs and water samples for laboratory diagnosis and tested water samples for microbiological quality. We identified 921 cases and one death among 8,367 residents (attack rate: 11%, case-fatality: 0.1%). The attack rate was the highest among persons of 50 years and above (14%) and females (12%). The outbreak started on 6 January and peaked on the 9th and lasted till 14 January. Cases were clustered around two major leakages in water supply system. Nine of the 16 stool samples yielded *V. cholerae* O1 Ogawa. We identified that consumption of water from the public distribution system (MOR=37, 95% CI 4.9-285, PAF: 97%), drinking unboiled water (MOR=35, 95% CI 4.5-269, PAF: 97%), and a common latrine used by two or more households (MOR=2.7, 95% CI 1.3-5.6) were independently associated with cholera. Epidemiological evidence suggested that this outbreak was due to ingestion of water contaminated by drainage following rains during cyclone. We recommended repair of the water supply lines, cleaning-up of the drains, handwashing, and drinking of boiled water.

## INTRODUCTION

Increase in diarrhoeal diseases following natural disasters of various kinds has been recognized worldwide (1,2). Major natural disasters, such as floods, tsunamis, earthquakes, tropical cyclones (e.g. hurricanes and typhoons), and tornadoes, could exacerbate the risk factors for transmission of infectious diseases by affecting pre-existing poor water, sanitation, and sewage systems (3). In fact, the risk of diarrhoeal disease outbreaks following natural disasters has been reported to be higher in developing countries (2). Specifically during a cyclone, disruption of the water distribution system, inadequate hygiene, sanitation, and poor access to health services often increase the risk of cholera outbreaks in such settings (4–6).

Annually, it is estimated that nearly 3 to 5 million cholera cases occur globally, with 10,000 to 200,000 deaths (7). Cholera continues to be an important public-health problem in India, during 1997-2006, sixty-eight outbreaks of cholera were reported from the country, affecting more than 200,000 persons, with 823 deaths (7). Outbreaks of cholera have been reported in areas with piped water systems that suffer from breaks in quality system and maintenance, including lack of chlorination, resulting in cross-contamination with the nearby sewage system (8–11). Contamination of drinking-water due to disaster has been responsible for some of these incidents (2). Cholera affects both children and adults following ingestion of food or water contaminated with toxigenic bacterium *Vibrio cholerae* (12–14). The enterotoxin of the bacillus causes copious painless watery diarrhoea which can lead to severe dehydration and death. Two serogroups of *Vibrio cholerae* O1 and O139 are the commonest causes of outbreaks (12,13). They have a short incubation period ranging from two hours to five days, leading to explosive outbreaks (13). Eighty percent of the cases can be successfully managed with oral rehydration formulation alone (14,15). Effective control measures rely on the prevention, preparedness, and early mitigation response (14). Provision of safe water and sanitation is critical in reducing the impact of cholera and other waterborne diseases (14,15).

A severe cyclonic storm (named ‘Thane’ by the India Meteorological Department) that emerged from the Bay of Bengal hit the Southern India's east coastal region of Pondicherry on 30 December 2011 (16). It had the speed of 140 kilometres per hour and caused widespread flooding and uprooting of trees and power-lines. Subsequently, a week later, on 7 January 2012, a peripheral health facility reported clusters of cases with acute diarrhoea in two localities [population-size: 8,367] of the coastal district of Pondicherry. An investigation team comprising local health authorities, experts from local referral hospitals, and a public health trainee from central health services investigated this cluster to confirm the outbreak; characterize the causal agent; describe the outbreak by time, place, and person; identify the sources; and formulate recommendations for the control of outbreak.

## MATERIALS AND METHODS

### Descriptive epidemiology

To confirm the outbreak, we reviewed the monthly surveillance data for diarrhoea between January 2009 and January 2011. Further, we ascertained information regarding any recent population migration or changes in the surveillance system. We defined a case of diarrhoea according to the WHO guideline as the occurrence of more than three watery stools per day among the residents of affected localities between 6 and 18 January 2012 (17). The investigation team conducted active door-to-door search for cases in the affected localities and also stimulated passive surveillance in health facilities in the costal district to identify new cases. We collected personal history, including symptoms, from case-patients and established a line-listing. An epidemic curve was constructed to describe the development of the outbreak over time. We calculated the attack rate (AR) by age and gender, using population census available at the health centre as denominator. The cases were plotted on a map to understand the spatial distribution. To aid in generating hypotheses, we gathered information from case-patients, health workers, and local leaders, using an unstructured trawling questionnaire. On the basis of descriptive epidemiology and abovementioned interviews, we generated hypothesis about the possible sources of the outbreak.

### Laboratory procedure

We collected rectal swabs from patients admitted in government health facilities. We tested those by culture, antimicrobial sensitivity, and molecular characterization (18,19). The specimens were incubated in alkaline peptone water enrichment media for 6 hours. Enriched cultures were inoculated in thiosulphate-citrate-bile salts-sucrose (TCBS) agar for *Vibrio cholerae* and in *Salmonella* and *Shigella* agar for other enteropathogens. Subcultures were done after 18 hours at 37 °C on nutrient agar. Typical colonies were confirmed by standard biochemical and slide agglutination with *V. cholerae* serogroup O1 polyvalent antiserum, followed by Ogawa and Inaba antisera. Antimicrobial sensitivity was determined by the Kirby-Bauer disc diffusion method. Results were recorded as resistant, intermediate, or sensitive following the guidelines of the Clinical and Laboratory Standard Institute (CLSI). Further, genomic DNA was extracted and subjected to molecular analysis.

### Amplification of ***ctx***A gene of ***V. cholerae*** O1 strain

The fragment was amplified using the forward CTX2: 5′-CGGGCAGATTCTAGACCTCCTG-3’ and reverse CTX3: 5′-CGATGATCTTGGAGCATTCCCAC-3′ primers target for the toxigenic *V. cholerae* O1 strain (20). The amplification was carried out with 35 cycles of denaturation at 95 °C for 1 minute, annealing at 60 °C for 1 minute, and extension at 72 °C for 1 minute. Initial denaturation and final extension was done at 95 °C for 5 minutes and 72 °C for 10 minutes respectively. PCR products were separated on 1.5% agarose gel and analyzed in a gel documentation system (Bio-Rad, California, USA) (21).

### Purification and sequencing of ***ctx***A gene fragment

The PCR products were purified from the agarose gel by using Nucleospin @ Extract II kit (Macherey-Nagel, Germany) as per manufacturer's recommended protocol. DNA (200 ng of gel-purified product) was used with the Big Dye Terminator kit (version 3.1) (Applied Biosystem, Foster City, CA, USA) for the sequencing PCR. The template was purified and sequenced on a 3130xl Genetic Analyzer (Applied Biosystem, Foster City, CA, USA).

### Sequence analysis

The nucleotide sequences were matched by the BLAST search in the National Center for Biotechnology Information (NCBI) site for determining similarities with previously-reported sequences. The nucleotide sequence of the *ctx*A gene was compared with the *ctx*A orthologous sequences of other *Vibrio* species obtained from GenBank database. The *ctx*A gene sequences retrieved as above were subjected to multiple sequence alignment using ClustalW and in BioEdit software. Clonal analysis also has been done using Molecular Evolutionary Genetic Analysis (MEGA4).

Water samples taken from the affected area were tested for coliforms by membrane-filtration technique at the public health laboratory.

### Environmental investigation

The water supply pipelines and sanitation situation were reviewed. We checked water chlorination records and generator operations records at the pump-house providing head pressure to water-lines.

### Analytical epidemiology

To test our hypothesis on potential sources of infection and other risk factors, we conducted a matched case-control study in the area with high attack rate. For the purpose of the analytical study, we defined a case-patient as occurrence of acute watery diarrhoea of three or more stools per day in an individual aged above five years residing in the affected locality. We proposed an unmatched design and, thus, calculated a sample-size of 60 cases and 120 controls with assumptions of odds ratio (OR) of 2.5, power of 80%, and 95% confidence interval (95% CI). However, in the field, logistics permitted us to implement a matched design (22). We compared cases with controls matched for age (±2 years), gender, and neighbourhood. We defined a control as that without any signs and symptoms of diarrhoea during a reference period of three days preceding the illness in a case. We collected information on various potential exposures, using standardized, closed-ended questionnaire. For all the exposures, we used a reference period of three days preceding the illness. The potential exposures considered were: drinking-water, water-handling practices, food habits, and sanitation practices. We computed matched odds ratio (MORs) for discordant pairs and their 95% confidence intervals (CIs), using the Epi Info software (version 3.5.5) (CDC, Atlanta, GA, USA). We calculated the fraction of cases attributable to exposure in the population [population attributable fraction (PAF)], using the classical formula: {Proportion of cases exposed × attributable fraction among the exposed [(odds ratio-1)/odds ratio)]} (23).

### Confidentiality

We protected the confidentiality of participants through the use of codes. However, review of ethical committee did not apply as this was a public-health emergency response to an outbreak and was covered by normal practice (24).

## RESULTS

### Descriptive epidemiology

On the basis of surveillance data for the previous year, available with the primary health facilities and the health office of the district, we confirmed that the cluster of cases represented an unusual increase in the incidence of diarrhoea for January 2012 in the affected localities and the entire coastal district. Further, we identified that there was neither any influx of population nor any changes in the surveillance system in any of these localities during that period. Hence, the cluster of cases was considered an outbreak.

**Figure 1.**
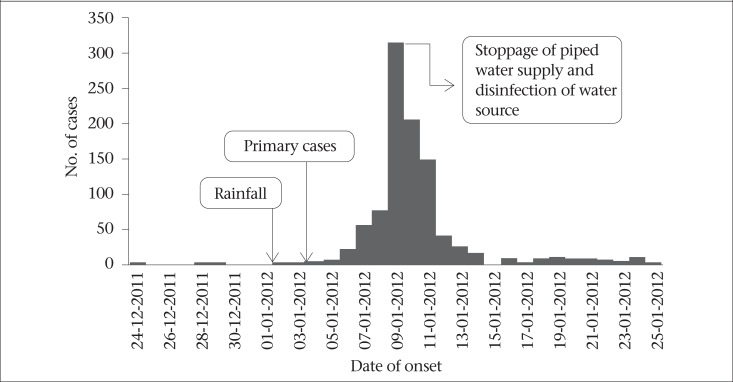
Distribution of diarrhoea cases by date of onset, Pondicherry, India, 2012

**Figure 2.**
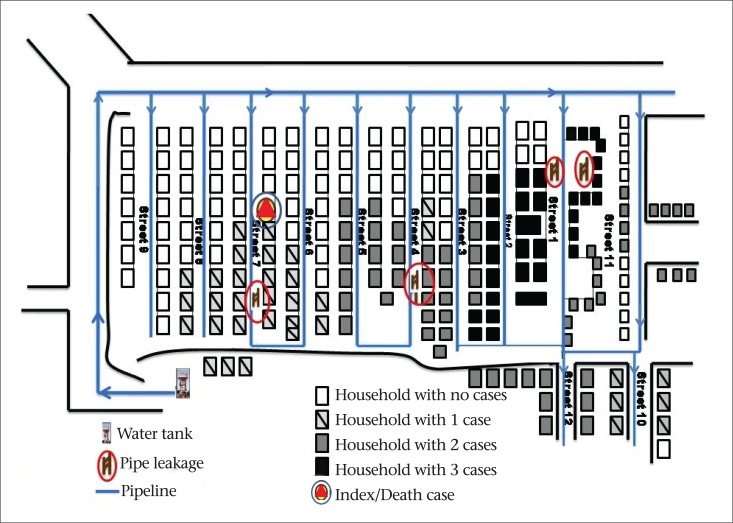
Spot map of diarrhoea cases by place of distribution, Pondicherry, India, 2012

We identified 921 case-patients among the 8,367 residents [attack rate 11%], with one death reported. The attack rate was higher among females and in the age-group of 50 years and above (Table 1). Nearly 50% of the case-patients had diarrhoea and dehydration; the remaining had a combination of diarrhoea, vomiting, with or without fever. The cases began appearing during the first week of January 2012, followed by a rapid increase to peak on 9 January and, thereafter, declined from 11 January with the last case reported on 14 January (Figure 1). Cases were clustered around two major breaks in the drinking-water pipelines (Figure 2).

**Table 1.** Attack rate of acute diarrhoea cases by age and gender, Pondicherry, India, 2012

Demographic characteristics		Cases	2011 population	Attack rate per 100,000
Age (completed years)	<5	76	762	9
	5-14	16	1,503	11
	15-49	475	4,681	10
	>50	202	1,421	14
Gender	Male	430	4,301	9
	Female	491	1,421	12
Overall		921	8,367	12

### Laboratory investigation

Nine of the 16 rectal swabs grew *V. cholerae* O1, serotype Ogawa biotype El Tor. All the strains were sensitive to tetracycline, norfloxacilin, ciprofloxacin, gentamycin, chloramphenicol but resistant to furazolidine, co-trimoxazole, nalidixic acid, streptomycin, and ampicillin. PCR analysis confirmed the biochemical identification and revealed the presence of virulence gene *ctx*A. The nucleotide sequence of the *ctx*A gene was compared with the *ctx*A orthologous sequences of other *Vibrio* species obtained from GenBank database, which confirmed the findings. The clonal analysis performed using MEGA4 (molecular evolutionary genetic analysis) demonstrated that a single clone of a *V. cholerae* strain was responsible for this outbreak.

### Environmental investigation

In the coastal district of Pondicherry, drinking-water is supplied through a series of overhead tanks. Each tank serves a locality with a network of pipelines. The main source of water is from the coastal aquifiers, where water is pumped into the overhead tanks. Prior to distribution, the water is tested and chlorinated. The water is distributed using pump-houses to maintain pressure in the pipelines. The supply is done thrice daily during specific times. The coastal district of Pondicherry is just four metres above the mean sea-level and, hence, it is served by a mix of drainage systems consisting of underground drainage in the town and septic tanks in other parts. On both sides of the roads, U-shaped drains carry sullage water. Although most of the houses have an individual connection of piped water, in some areas, a series of public water-taps in each street provides potable water to the residents. These connections cross the sullage water drains running on both sides of the streets. The localities that reported current cluster of cases did not have an underground drainage system.

Following the cyclone, there was flooding with stagnation of rain and sullage water in the drains. We observed two major leaks in the pipes connecting water distribution system and the public water-taps crossing the U-shaped drains (Figure 2). These leakages and broken pipes provide entry-points to the sullage-mixed rain water through suction or negative backflow. The generator records showed that it was fully functional during the cyclone and that the water was chlorinated as per the recommended procedures.

### Analytical epidemiology

We included 66 case-patients identified during door-to-door survey and 88 controls. The number of sets for which we matched cases-to-controls ratio was as follows: 50 sets for 1:1; 10 sets for 1:2; 6 sets for 1:3. Sixty-four (97%) of the 66 case-patients had consumed water collected from the public water distribution system compared to 40 (45%) of the 88 matched controls. We identified that consumption of water from the public distribution system (MOR=35, 95% CI 4.9-285), drinking unboiled water (MOR=37, 95%, CI 4.5-269), and the use of a common latrine by two or more households (MOR=2.7, 95% CI 1.3-5.6) were independently associated with the cases of cholera infection (Table 2). Calculation of the population attributable fraction for drinking-water from the public distribution system could explain 97% of the case-patients, and that for other risk factors, such as drinking unboiled water and the use of common latrine by two or more households, was 97% and 50% respectively.

**Table 2.** Frequency of selected exposures among acute diarrhoea cases and controls, matched case-control study, Pondicherry, India, 2012

Risk factor	No. (%) of case-patients (N=66)	No. (%) of controls (N=88)	Matched odds ratio (95% CI)
Consumption of water from the public drinking-water system	64 (97)	40 (45)	37.0 (4.0-285.0)
Not boiling water	64 (97)	44 (50)	35.0 (4.0-269.0)
Use of common latrine	35 (53)	26 (29)	2.7 (1.3-5.6)

## DISCUSSION

In India, cholera epidemic is generally linked with the monsoon and contamination of drinking-water either due to sewage or polluted water bodies. Lack of proper arrangement for safe drinking-water caused increased prevalence of the disease (25). Under similar conditions, two contiguous localities in the costal district Pondicherry were affected with a cholera epidemic, following the torrential downpour due to cyclone Thane in January 2012. Both epidemiological and environmental evidences suggest that this outbreak could be due to consumption of drinking-water contaminated by sullage.

The striking feature in this outbreak is that all age-groups were affected. The attack rate increased with age. The finding in this study suggests that the risk of exposure and vulnerability to infection is uniform across all age-groups. The attack rates observed in community settings ranged from 0.3 to 7% (26–28). Outbreaks with attack rates exceeding 5% have been reported in refugee-camp setting and resource-poor settings (29,30).

The investigation suggests that the outbreak might have been caused due to contamination of water distribution pipelines by the suction or negative backflow through leaking and broken pipes, following rise in the levels of sullage-mixed rainwater while crossing the storm-water drains. Majority (97%) of the cases in the population could be attributed to the consumption of water from the contaminated source from the public water distribution system. Similar observations were made during cholera outbreaks following cyclone ‘Aila’ and earthquake in Haiti (31,32). Corrosion, cross-connections, backflow, improperly-protected storage or repairs to water mains and plumbing, etc. are other major reasons for cholera outbreaks (33). These observations hold well for the current outbreak. The United Nation's Millennium Developmental Goal (MDG) aims to reduce the proportion of population without access to safe water and sanitation to decrease to half by 2015 (34). While the MDG considers piped water as an improved water source, transmission of waterborne pathogens may occur when pipes are not periodically maintained. This study confirms the earlier reports which have underlined the importance of periodic maintenance of the water pipelines. The other risk factors that were found to be significant were: not boiling water (97%) and the use of common latrine (50%). Studies from slums of Kolkata and Peru have reported that consumption of unboiled water appeared to be the single-most important risk factor for illness (35–38). Boiling is, sometimes, impractical because of high cost or non-availability of fuel; this was relevant to this outbreak because anticipated short supply of cooking-gas (liquefied petroleum gas) prevented the people from consuming boiled water. Meanwhile, the residents who consumed bottled water remained unaffected. Sharing a common latrine by two or more households in a house is common in low-income settings in India and resource-poor settings elsewhere. In this study, it was found to be a significant risk factor. Similar situations where multiple households use the same latrine provide additional opportunities for faecal-oral transmission of cholera (29,30,39).

### Strengths and limitations

Our study had three limitations. First, the case-control study was conducted in a highly-affected area during the outbreak period; so, we had first proposed to do an unmatched study, then did a matched design; this reduced the sample-size available for the analytical study but our study had sufficient power to detect the association between the illness and drinking-water (power 80%). Second, we did not test the status of subclinical infection among controls. This would have amounted to a non-differential misclassification. This error would only lead to an underestimation of the strength of the association, which would not affect our conclusion. Third, in a disaster, people tend more to blame ‘systems’ (in this case public water supply) than behavioural issues. We had behavioural risk factors emerging as significant in this study.

Our investigation pointed to the direction of how a natural disaster, like cyclone, can lead to the spread of infectious diseases, like cholera, following contamination of public water distribution system by the drainage system. Consumption of contaminated water led to a common source cluster, followed by possibility of a certain degree of person-to-person transmission. Molecular analysis proved that a single clone of toxigenic *V. cholerae* O1 biotype El Tor was the causative agent, highlighting the need for continuous monitoring to facilitate early interventions against any potential epidemic by this biotype.

### Conclusions

We formulated a number of control measures. The short-term measures were initiated with cessation of water distribution to repair breaks and leaks followed by flushing of water distribution systems and super-chlorination. Safe water was provided using water-tanks and distribution of water cans. Medical camps were set in the affected localities and dedicated wards in hospitals to deal with cholera cases. Intense education campaigns, like importance of boiled water consumption and sanitation, were carried out. Long-term preventive measures included phased construction of underground drainage system and relaying of water pipelines and domestic connections to run over the closed drainage lines.

## ACKNOWLEDGEMENTS

We sincerely acknowledge the Director of Medical Services, Pondicherry, for his permission to conduct the investigation. The National Institute of Epidemiology (ICMR) and ICMR School of Public Health supported this work as an intramural project.

**Conflict of interest:** Authors report no conflicts of interest.
